# A severe refractory COVID-19 patient responding to convalescent plasma; A case series

**DOI:** 10.1016/j.amsu.2020.06.018

**Published:** 2020-06-24

**Authors:** Hadi Mohammed Abdullah, Hersh H. Hama-Ali, Sabah Nasraddin Ahmed, Kosar Muhammad Ali, Kamaran Amin Karadakhy, Safeen Othman Mahmood, Zana Hameed Mahmood, Karmand Qadir Hamad Amin, Peshnyar Muhammad Atta, Bryar Ezadeen Nuradeen, Shvan H. Mohammed, Rawezh Q. Salih, Hiwa O. Baba, Fahmi H. Kakamad

**Affiliations:** a Shar Hospital, Sulaimani, Kurdistan, Iraq; b Shahid Dr.Hemn Hospital, Sulaimani, Kurdistan, Iraq; c General Director of Health in Sulaimani, Kurdistan, Iraq; d College of Medicine, University of Sulaimani, Sulaimani, Kurdistan, Iraq; e Molecular Diagnostic Laboratory, Sulaimani Veterinary Directorate, Sulaimani, Kurdistan, Iraq; f Central Public Health Laboratory, Sulaimani, Kurdistan, Iraq; g Kscien Organization, Hamdi Str, Azadi Mall, Sulaimani, Kurdistan, Iraq; h Smart Health Tower, Sulaimani, Kurdistan, Iraq

**Keywords:** COVID-19, SARS-CoV-2, Coronavirus, Convalescent plasma

## Abstract

**Introduction:**

Although some medicines are under research, currently, no specific antiviral drug has been approved to target 2019 novel coronavirus. In this report two severe cases of 2019 novel coronavirus disease (COVID-19) patients have been described who received convalescent plasma (CP).

**Case report:**

Two male cases (a 46-year-old and a 56-year-old) after being diagnosed with severe COVID-19, they deteriorated despite supportive care and antiviral therapy. They started to improve with CP infusion both clinically and radiologically. Finally they were discharged in a very well condition with negative virology tests.

**Conclusion:**

CP might be an effective therapy for severe COVID-19 patients.

## Highlights

• Currently, no specific antiviral drug has been approved to target novel coronavirus.
• Convalescent plasma is a classical immunotherapy.
• It has been used in the management of several infectious diseases for many decades.
• In this report two severe cases of COVID-2019 have been described receiving CP.

## 1 Introduction

In the last few months, a disease caused by severe acute respiratory syndrome coronavirus 2 (SARS-CoV-2), called COVID-19 has spread worldwide. It was declared to be a pandemic on March 11, 2020 by World Health Organization (WHO) [1,2]. About 14% of the cases develop severe disease and 5% require admission to intensive care unit [3]. Although some medicines are under research, currently, no specific antiviral drug has been approved to target SARS-CoV-2 [1,2]. Moreover, giving corticosteroid to a patient with lung injury caused by COVID-19 remains controversial [4]. Owing to the absence of specific antiviral drugs, it is wise to search and try other alternative strategies to treat COVID-19. CP is a classical immunotherapy which has been used in the management of several infectious diseases for many decades. It was fruitfully used in the treatment of 2009 H1N1, SARS, MERS [[5], [6], [7], [8]]. A meta-analysis enrolling 32 researches of severe influenza and SARS coronavirus infection revealed a significant dropping in the mortality after giving CP compared with the control groups [9]. CP therapy, however, was incapable to improve the outcome in the patients with Ebola virus infection [10]. Authors thought that because of the similarity among MERS, SARS, and COVID-19, CP therapy could be a viable option for COVID-19 patients [11].

The aim of this paper is to report and discuss two cases of severe COVID-19 who responded very well to CP therapy while they were refractory to the other lines of management. The article was reported in line with PROCESS guidelines [12].

## 2 Methods

### 2.1 Registration

The study registry has been provided in accordance with the declaration of Helsinki–“Every research study involving human subjects must be registered in a publicly accessible database before recruitment of the first subject”. The research was registered in the Chinese Clinical Trial Registry. The registration number is ChiCTR2000033323 (http://www.chictr.org.cn/hvshowproject.aspx?id=35675).

### 2.2 Setting

The patients were managed in the governmental hospitals. The procedure was supervised by the first author and shared directly by the first 10 authors.

### 2.3 First case

A 46-year-old man presented with mild cough and fever for 4 days. He was a known case of hypertension and had history of contact with suspicious cases of SARS-CoV-2. Clinical examination and vital signs were normal apart from low grade fever (temperature: 38.2C).

**Fig. 1:**
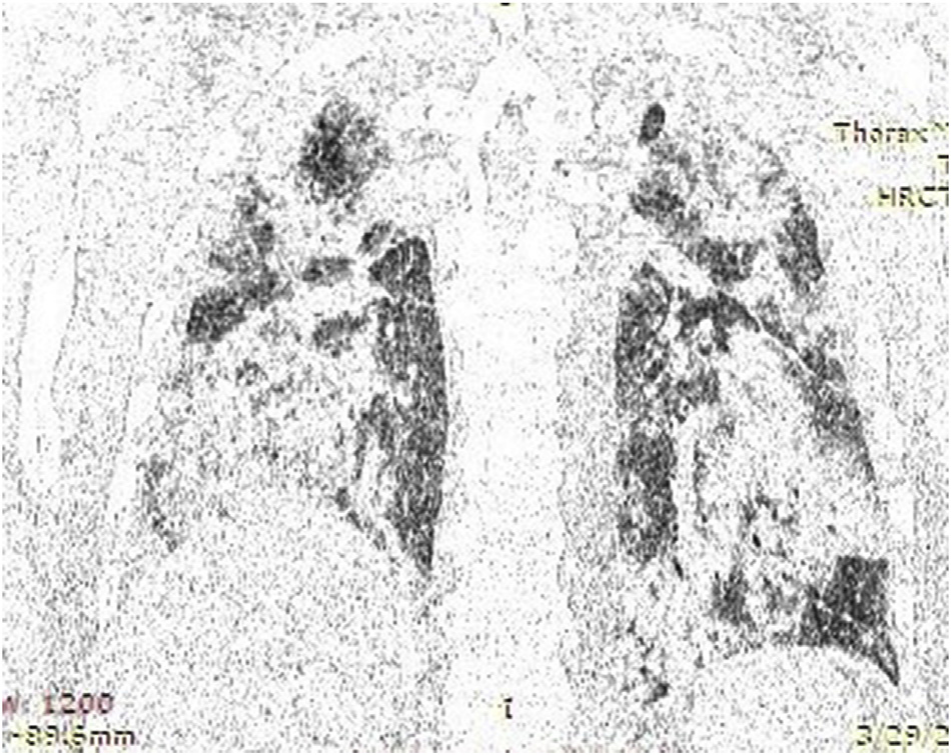
CT-scan (coronal section) showing diffuse ground glass opacity with multiple areas of consolidation.

Chest –X-ray was normal. Hematological tests showed lymphopenia, high (58) erythrocyte sedimentation rate (ESR). Real Time-Polymerase Chain Reaction (RT-PCR) for nasopharyngeal swab was positive for SARS-CoV-2, serum ferritin was 746 ng/ml. The patient was admitted in the corona isolation unit center (CIUC). He received Hydroxychloroquine (400 mg b.i.d) and Azithromycin (500 mg q.d.). The patient's condition progressed, on the second day, he developed dyspnea (respiratory rate: 35 breaths/minute), oxygen saturation was 80%, temperature: 39.5 C, arterial blood gases (ABG) on room air showed PaCO2: 23 mmHg, PaO2: 57 mmHg, PaO2/FiO2: 114 mmHg. Chest x-ray showed right upper zone ground glass opacity with small area of consolidation. Computed tomography (CT) scan showed small subpleural ground glass opacity (GGO) affecting both lower lobes and right upper lobe. The patient was put on noninvasive oxygen therapy, Meropenem vial (1 g t.i.d.), Hydroxychloroquine (400 mg b.i.d) and Kaletra tablet (Lopinavir/Ritonavir 800/200, b.i.d), and enoxaparin (4000 IU q.d.). After 3 days, the patient did not respond to the management strategy and deteriorated more and more. The dyspnea increased, (Oxygen saturation 60% without O2, became 90% on 10 L O2 through nasal cannula), bilateral diffuse course crackles, temperature 39.5 C, ABG on 10 L O2 through nasal cannula showed PaCO2: 33 mmHg, PaO2: 58 mmHg, PaO2/FiO2: 96 mmHg, serum ferritin: 1074 ng/ml, ESR: 91, D-dimer 1140 ng/ml, C-reactive protein (CRP): 37 mg/dL, with normal troponin test. CT scan showed diffuse bilateral GGO and multiple areas of consolidation in different regions of the entire lung (Fig. 1). Despite his treatment, the patient received 200 ml of CP from a previously recovered moderate COVID-19 patient after performing necessary investigations for donor plasma (hemoglobin level and viral screen). The patient started to improve clinically, 4 days later, he was quite stable, no dyspnea, oxygen saturation on room air reached 95%, and the chest x-ray resolved partially. The patient was discharged from the hospital 16 days after admission in a healthy condition without symptoms, chest examination was clear, no significant radiological findings on chest-x-ray, and there were three consecutive negative RT-PCR tests each with at least 24 h apart.

### 2.4 Second case

**Fig. 2:**
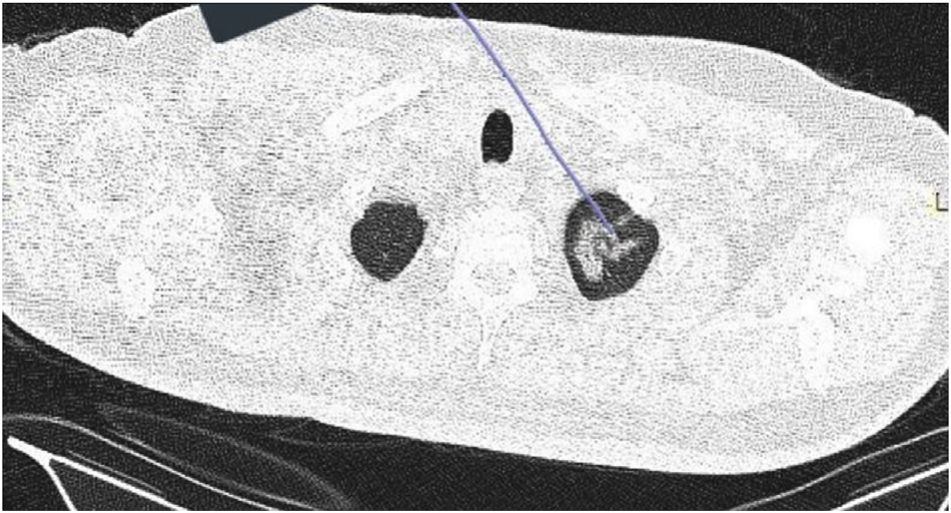
Chest CT-scan (axial section) of the second case showing bilateral diffuse ground glass opacities with consolidation.

A 56-year-old male patient presented with flu-like illness with high fever for three days. He was a known case of hypertension controlled by Valsartan tablet. He was a shopkeeper denying history of both contact and travel. The patient was admitted and put on antibiotics and antipyrol after investigation. Hematological tests and chest x-ray were normal, RT-PCR for nasopharyngeal swab was negative despite increase in severity of cough, shortness of breath, and fever. Four days later, RT-PCR became positive for SARS-CoV-19. The patient condition progressed, on sixth day of hospitalization, he had dyspnea, fever, cough, tachycardia and hypoxia with diffuse bilateral crepitation on chest examination; oxygen saturation: 80% increasing to 90% on 8 L of oxygen, respiratory rate 40 breaths/minute, temperature 39.5C, PaO2: 61 mmHg, PaO2/FiO2:122 mmHg on 10 L O2 through nasal cannula, alveolar-arterial gradient: 65. CBC showed lymphopenia, serum Ferritin: 650 ng/ml, CRP: 45 mg/dL, ESR: 91. Chest-x-ray revealed bilateral lower zone infiltration and haziness, chest CT scan showed left upper lobe ring shaped consolidation surrounding small area of GGO (reverse Halo sign or Atoll sign), bilateral pleural effusion and bilateral diffuse lower lobe GGO (Fig. 2). The patient was put on non-invasive oxygen therapy, Meropenem vial 1 gm t.i.d., Tamiflu tablet 75 mg b.i.d., Kaltetra tab 400/100 mg b.i.d., hydroxychloroquine tablet 400 mg b.i.d. for the first day, and 200 mg b.i.d. subsequently, Enoxaparin 4000 IU q.d. subcutaneously, acetaminophen bottle on need, Valsartan tablet was replaced with Amlodipine tablet. Within the four subsequent days, the patient deteriorated, oxygen saturation decreased to 70–80% on 10 L of oxygen, PaO2: 56 mmHg, PaO2/FiO2: 93, alveolar-arterial gradient: 75; D-dimer:1992 ng/ml, serum Ferritin: 692 ng/mLn, CRP: 93 mg/dL, ESR: 110, with normal renal function tests, liver function tests, echocardiography troponin test and serum electrolytes. In addition to his medications, the patient received 200 ml of CP from a previously recovered COVID-19 patient after doing necessary investigations for donors plasma (hemoglobin level and viral screen). Seventy-hours later, the patient started to improve, fever subsided, dyspnea decreased, lymphocyte counts normalized. Six days after the plasma transfusion, D-dimer 1887 ng/ml, PaO2: 81 mmHg, PaO2/FiO2 = 162 mmHg on 10 L of nasal cannula oxygen. Chest x-ray showed improved consolidation and GGO, CT-scan showed disappearance of the pleural effusion. The patient was discharged home 21 days after the admission with good health, he had three consecutive negative RT-PCR each 24 h apart.

## 3 Discussion

According to WHO guidelines, the main strategy in the management of COVID-19 is prevention of the infection, early recognition of the cases, isolation, monitoring, and supportive care. Immunoglobulins or CP from the recovered patients have been given to the patients with severe COVID-19 as a last choice to revert the progress of the disease despite supportive treatment [13].

Although WHO recommended extracorporeal membrane oxygenation (ECMO) to support appropriate patients with acute respiratory distress syndrome (ARDS), its use is restricted to the specialized centers [14]. In the management of these two cases, ECMO was not an option because of facility shortage in the center.

A retrospective study by Zhou and colleagues which included 72,314 patients infected with SARS-CoV-2 revealed that 5% of them had life-threatening conditions, and nearly half of the cases had comorbidities, most commonly diabetes and cardiovascular diseases [15]. Both of the current cases had history of hypertension.

Even with the potential efficacy of CP, there have been occasional rigorous efforts to transfuse it as a preliminary therapy against this pandemic communicable threat. The lack of well-conducted clinical trials undoubtedly contributes to the uncertainty to practice this management strategy. Moreover, the most active formulations (hyperimmune globulin, H-Ig, or CP) are unidentified. CP has the benefit that while it halts the viral replication by its antibodies, other components can likewise apply valuable effects such as refilling of the consumed coagulation factors. The problem with CP is that it demonstrates individual dependent variableness in antibody titers and specificities while H-Ig preparations hold standardized doses of antibody [2]. However in the current cases, we were obliged to use CP because of the absence of H-Ig in the center.

Just few days before our intervention, US Food and Drug Administration (FDA) approved CP from those individuals who have already recovered from the disease stating that they might hold active antibodies against the virus. It is recommended to be used in severe and life-threatening conditions. Severe disease is a clinical situation in which the patient has dyspnea, tachypnea (respiratory rate ≥30 breaths/minute), oxygen saturation ≤93%, PaO2/FiO2 <300, or >50% lung infiltrate on chest x-ray. Life threatening disease is recognized when there is septic shock, respiratory failure, multiple organ failure or dysfunction [16].

Shen and associates published their experience in a pilot study including five severely diseased patients with COVID-19 using CP from the cured individuals. The patients complained from severe respiratory failure requiring mechanical ventilation; two patients had fungal and/or bacterial pneumonia and one case received ECMO. The CP showed IgG and IgM anti–SARS-CoV-19 antibodies and showed the capacity to deactivate the virus in the cultures. Although the patients were kept on the antiviral drugs (lopinavir/ritonavir), the authors contributed the CP to the recovery of the patients since they responded seven days after plasma transfusion. The body temperature normalized and the organs recovered. Twelve days after the transfusion, the respiratory samples became negative for the virus [17]. In the current cases, the plasma was not tested against SARS-CoV-19 in a culture to show effect of the plasma in vitro due to lack of the required resources.

Both of the current cases were not put on mechanical ventilation as the authors think that in most of the time intubation in COVID-19 patients spreads the infection and worsens the condition.

In conclusion; CP might be an effective therapy for severe COVID-19 patients. No serious adverse effect was observed in the current cases during and after the transfusion. However, the influence of supportive care and patient's immune response couldn't be shown. A well-designed trial is required to determine the efficacy and safety of CP in patient with severe COVID-19.

## Patients' consent

A written informed consent has been taken for publication of this report. Provenance and peer reviewed: Not commissioned, externally peer reviewed.

## Declaration of competing interest

None to be declared.
